# Binder Jetting 3D Printing of Binary Cement—Siliceous Sand Mixture

**DOI:** 10.3390/ma17071514

**Published:** 2024-03-27

**Authors:** Mursaleen Shahid, Vincenzo M. Sglavo

**Affiliations:** Department of Industrial Engineering, University of Trento, 38123 Trento, Italy

**Keywords:** quick-setting cement, Portland cement, concrete 3D printing, binder jetting

## Abstract

Three-dimensional printing allows accurate geometries to be obtained across a wide range of applications and it is now also moving into the architecture and construction industry. In the present work, a unique binary mix composed of ordinary Portland cement (OPC) and quick-setting cement (QSC) was combined with silica sand aggregate in different proportions for a customized binder jetting 3D printing (BJ3DP) process. Specimens were printed using the blended dry powders and deionized water to determine the impact of the processing variables on the properties of the realized specimens. The results show that the properties are influenced by the binary mix proportions and the layer thickness. The investigation found significant improvement in mechanical performance on increasing the proportion of OPC and optimal conditions were identified with proportions of 35 wt% OPC and 5 wt% QSC. Notable enhancements were also observed as the layer thickness was reduced.

## 1. Introduction

The early development of 3D printing technology, commonly referred to as additive manufacturing (AM), began in the early 1980s [1]. This technique has had a significant impact across numerous technologies [2,3,4,5,6,7,8]. Apart from the type of AM techniques as per ASTM standards [9], the primary benefit of AM is its capacity to convert complex shapes from CAD files into physical prototypes [10]. Its usage for concrete printing has reduced the cost by up to 60% based on the removal of formwork [11,12]. Robocasting and binder jetting are the two most adaptable techniques of AM in the field of construction, the former being mainly categorized in terms of material extrusion-based printing techniques [13], where interfacial porosity and low printing accuracy represent some specific drawbacks [14].

Binder jetting 3D printing (BJ3DP) technology was first proposed in 1993 at MIT [15,16,17] and it has some additional benefits compared to robocasting. The main advantage is that the material around the printed shape acts as a support [18] and, therefore, no additional scaffolds are required. Additionally, the removal of the unbounded dry powder is easier for large, printed bodies and this technique is more appropriate for high scalability [19]. In said 3D printing technique one starts with the build platform, which is carefully covered with a fine layer of powder; particle distribution, size and shape have an impact on the powder bed, which is important for the later stages [20,21,22]. Subsequently, a liquid binder is selectively sprayed onto the powder layer by a precision inkjet printhead, which binds the particles together in line with the digital design. The powder bed is then lowered to facilitate the spread of the next powder layer and the process continues layer by layer until the entire body immersed in unbound powder is formed [23,24,25,26,27,28]. The finished geometry is then carefully separated from the unbound powder using a vacuum or a brush [29,30,31].

The first binder used in BJ3DP technology was calcium sulphate hemihydrate (generally referred as gypsum or plaster) [32]. The work of Zhou et al. mainly describes the procedure and process parameters of powder-based printing. Their findings conclude that fine powder is the general choice for high-resolution parts. Nevertheless, the choice of layer thickness is mainly dependent on the maximum aggregate size.

The BJ3DP process relies on the flowability of the powder, which is a critical parameter. Maintaining a consistent flowability is essential for achieving the desired outcome. A one-dimensional powder representation can be used to concisely describe flowability and provide a scale for grading powders accordingly. Powder flowability is typically measured using several parameters and methods, such as the Hausner ratio, angle of repose, flow through an orifice, Carr’s compressibility index, the shear cell method and the cohesion index. These methods, which have been widely referenced in the literature [33,34,35], provide a comprehensive understanding of the various facets of powder behavior in various contexts. Powders with low flowability can reduce the resolution of the printed item, while high flowability powders help to improve resolution in the finished printed product. Among other parameters, the Hausner ratio and Carr’s index are vital reference points for describing the powder’s flow characteristics. In general, Hausner ratio values less than 1.25 indicate good to exceptional flowability, while values more than 1.25 may indicate flowability issues. Carr’s index values are also thought to indicate excellent flow when they are less than 15% and impaired flowability when they are larger than 25%. These specified ranges aid in the development of an extensive framework for assessing the powder’s flow properties.

The type of cement used has a major impact on the density and mechanical strength of 3D-printed concrete specimens; in addition, changes in density are linked to variations in porosity. Notably, variations in porosity result from the proportion of air-filled gaps, which affects the weight of the concrete. The density of concrete, on the other hand, is determined by the specific gravity of its aggregate, as well as the qualities of the other components. These factors highlight the complex interplay between the mechanical characteristics of 3D-printed concrete structures, cement type and perceived density.

The goal of improving the physical and mechanical properties of the printed material can be achieved by optimizing the process parameters [36]. For all types of additive manufacturing processes, the layer thickness is the printing parameter that matters most [37]. When considering the thickness of a deposited layer, it has a significant impact on the characteristics of the printed items including strength, printing time and quality of the printed geometry [10,38,39,40,41]. The calculation of layer thickness is intricately tied to the resolution of the printing component and particle size [42]. Yu et al. [43] investigated the void size distribution and mechanical performance of 3D-printed concrete using mercury intrusion porosimetry (MIP) and X-ray computed tomography (CT). The obtained results show that 3D-printed materials have more macropores and large voids, which are related to printhead movement, decreased vibration and quick moisture loss. By comparing these specimens to mold-cast ones, the void morphologies are noticeably more irregular and extended, especially between layers, and this results in decreased strength. The time elapsed between successive printing layers affects the air voids in concrete [44]. Xin et al. [45] focused on a modified cement combination made up of ordinary Portland cement (OPC) and rapid-hardening sulpho-aluminate cement. According to their work, specimens produced at lower velocities have larger layer thicknesses and overall increased height. Using the extrusion-based technique (robocasting), Nair et al. [46] examined the layer thickness effect on the mechanical properties of fiber-reinforced 3D-printed beams. Their work concluded that thinner layers are more beneficial for 3D-printed mortars. Ur Rehman et al. [47] explained that the geometrical and mechanical properties of OPC-based materials depend on the specific binder used [48].

The use of binary cement in 3D printing has been shown to be a potentially useful approach to improve mechanical properties. Soltan et al. [49] processed a composite using a modified dry mixture of calcium aluminate cement, fly ash and ordinary Portland cement. The blend included aggregates of different kinds of silica, superplasticizer admixtures and viscosity modifiers such as high-performance methylcellulose (HPMC) and nano-clay. Furthermore, 2 vol% polyvinyl alcohol fibers were incorporated. Extrusion with a manually controlled caulk gun was used and the resulting printed specimens showed failure strains between 2 and 4% and tensile strength between 2 and 4 MPa. Shakor et al. [50] also worked on the same binary cement mix of OPC and calcium aluminate cement along with Li_2_CO_3_ as an activator to reduce the setting time; the maximum compressive strength obtained was 14.68 MPa after 28 days. In another work [51], they emphasized the impact of heat curing on the mechanical properties of the same modified binary cement. In a study by Anderson et al. [52], four different kinds of plasters and particulate materials were analyzed, incorporating an aqueous solution composed of 95% water and 5% humectant and glycerol; potassium sulfate was also added as an accelerator.

In another work, Gibbons et al. [53] selected a superior composition consisting of 97 wt% rapid-hardening cement and 3 wt% polyvinyl alcohol. The researchers tested the material’s performance using different curing procedures in water at room temperature and at 80 °C. The results were based on different saturation levels for the shell and the core. Their analysis showed that flexural strength decreased because of a reduction in core saturation level. This thorough investigation emphasizes how curing circumstances crucially affect the resulting material’s mechanical properties. Similarly, Zhou et al. [54] explored the 3D printing of various powder mixtures containing calcium phosphate and calcium sulphate in a water-based binder. Their goal was to understand the interactions between many factors, such as the wettability of the powder binder, the behavior of the binder drop penetration, the packing of the powder bed during the process and the final quality of the printed samples. Their results emphasized how crucial calcium phosphate particle size and calcium phosphate/sulphate ratio are as critical elements affecting the 3D printing process. The application of magnesium ammonium phosphate, or struvite, in a neutral setting reaction was investigated in another study [55]. Klammert et al. printed farringtonite (Mg_3_(PO_4_)_2_) powder using an ammonium phosphate solution as binder, obtaining a compressive strength ranging from 2 to 7 MPa (290 to 1015 psi). In a different method, Shakor et al. [56] used gypsum hemihydrate in place of cement mortar powder, allowing the resultant parts to be used in building applications.

The present study aims to identify the most effective composition of a binary cement mixture by considering its final mechanical properties and evaluate how the layer thickness during BJ3DP influences them.

## 2. Materials and Methods

The starting powder used for the BJ3DP process was composed of quick-setting cement (QSC) from Vicat cement—OBI Italy, ordinary Portland cement, OPC (Tecnocem 32.5, Heidelberg Materials, Peschiera Borromeo, Italy) and siliceous sand (grade 0.4–1.18 mm) from Sabbie di Parma (Polesine Zibello, Italy) in different proportions as shown in Table 1. Each composition was carefully mixed in a concrete mixture for 30 min to guarantee homogeneity of the dry mixture. Deionized water was used as liquid binder. Both OPC and QSC are hydraulic binding materials, where OPC exhibits standard setting time and consists mainly of Portland cement clinker, the appropriate amount of gypsum and some percentage of blended materials. Quick-setting cement is formulated to take less time to set and harden when compared to Portland cement. This strategic blend aims at balancing the strength development and setting time of the 3D-printed specimens.

**Figure 1:**
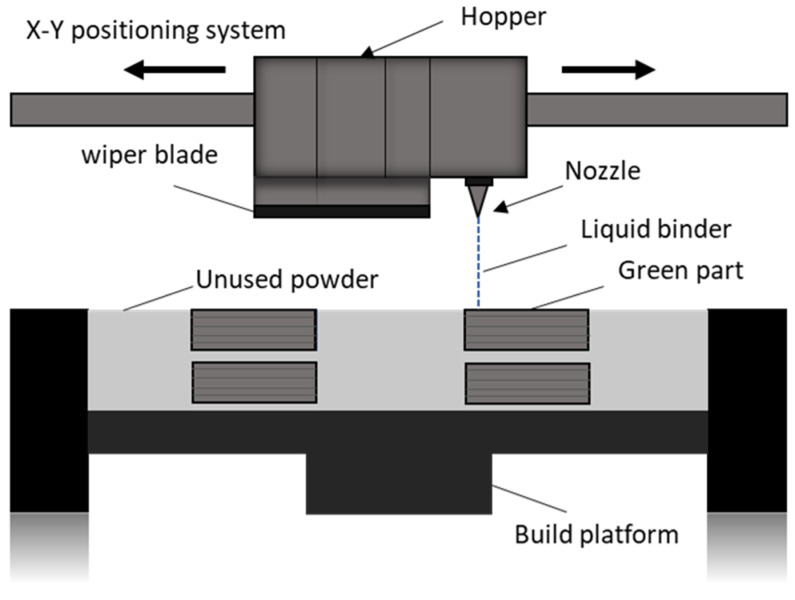
Schematic of 3D printing machine.

A customized 3D printer was employed to produce concrete-based specimens through a sequential layering process. A schematic of the customized 3D printer is shown in Figure 1. 

The printer was equipped with pressurized opening and closing of a hopper for the dry powder supply and a liquid binder dispensing system. The dry powder was deposited on the powder bed in the X direction where a wiper blade allowed uniform distribution of the powder on the bed. A Staiger valve along with a nozzle of internal diameter 0.19 mm by Lee Co. (Westbrook, CT, USA) was used for a drop-on-demand supply of the water. The pressure was controlled by the system’s precision valve to provide the required amount of binder and these values were monitored and recorded by a pressure sensor. The printed portion was consolidated in the areas where the dry powder and water came into contact, with the unaffected dry powder acting as a supporting material around the printed portion. Printed parts underwent controlled curing for 24 h within the powder bed in the lab atmosphere. Subsequently, they were extracted from the powder bed and the excess dry powder attached to the printed surface was carefully removed. The printed specimens were placed under a hood to cure at a temperature of 20–25 °C while maintaining a humidity of 50%.

The specimens’ CAD model was created using SolidWorks, to obtain geometries suitable for flexural and compressive strength testing, and it was then sliced to generate a G-code script that was associated with each printing batch. The details of the 3D printing process were defined by the G-code program, which contained parameters like hatch distance, feed rate, printhead height from the powder bed and layer thickness.

The hatch distance and the printhead height from the powder bed were maintained at 2 mm and 10 mm, respectively, and the layer thickness was varied from 1 mm to 2 mm. To ensure stable water–cement ratios and similar outcomes for varied layer thicknesses, the feed rate was regulated within the range of 7.2 to 14.0 m/min [57].

The Hausner ratio and Carr’s index are discussed in this paper to describe the flowability of the mixtures. The Hausner ratio (HR) is calculated by dividing the tapped density ($\rho_{t}$) by the bulk density ($\rho_{b}$) as:

(1) $$HR=\frac{\rho_{t}}{\rho_{b}}$$

Carr’s index, which expresses the material compressibility, is calculated using the following relation:

(2) $$CI=\frac{\rho_{b}-\rho_{t}}{\rho_{b}}\times 100$$

Notably, Hausner ratios below 1.25 imply exceptional flowability, whilst values beyond this cutoff can suggest possible flowability problems. Comparably, values of Carr’s index that are less than 15% indicate optimal flow, whereas values that are more than 25% may indicate poor flowability. The evaluation criteria used in this study are compatible with accepted standards and procedures specified in previous research [34].

The apparent density of the printed specimens was measured in accordance with ASTM C 642 norm [58]. The specimens were first oven-dried for at least 24 h at 110 °C. They were then immersed in water for 48 h and boiled for 5 h in a beaker on a hot plate and cooled for 3 h by immersion in water. Following the steps specified in the cited norm, the mass of the specimens was carefully reported at every stage.

Standardized parameters described in the EN-196-1 norm were carefully followed in the creation of specimens for both three-point bending and compressive strength tests [59]. Specimens of dimensions 160 mm × 40 mm × 40 mm and 40 mm edge cubes were printed for flexural and compressive strength testing, respectively.

The mechanical tests were carried out using a universal testing machine (Model 810, MTS System, Eden Prairie, MN, USA). Bending tests were performed with a rate of 50 N/s. The flexural strength was calculated as:

(3) $$f_{f}=\frac{3FL}{2bd^{2}}$$

where *F* is the failure load, *L* the span length (140 mm), and *b* and *d* the specimen width and thickness, respectively.

Compression tests were performed with a rate of 2400 N/s and the compressive strength was determined as:

(4) $$f_{C}=\frac{F_{C}}{A}\;$$

where *F_C_* is the failure load and *A* the specimen cross-sectional area. Here the constant loading rate was kept at 2400 N/s.

SEM analysis was conducted on seven-day cured specimens printed with layer thicknesses of 1 mm and 1.5 mm. Carefully polished specimens were used for the SEM analysis. The samples were dried in an oven set at 105 °C. They were carefully cleaned with ethanol and coated with a nanometric layer of Pt–Pd alloy to make them electrically conductive so as to be suitable for analysis by SEM (JEOL, JSM5500, Frenchs Forest, NSW, Australia) according to the ASTM C 1723 [60] norm.

The lateral surface of the 3D-printed specimens was also examined using a digital microscope (DSX 1000, Olympus, Segrate, Italy). After seven-day curing, specimens printed with layer thicknesses of 1 mm and 2 mm were analyzed.

## 3. Results

**Figure 2:**
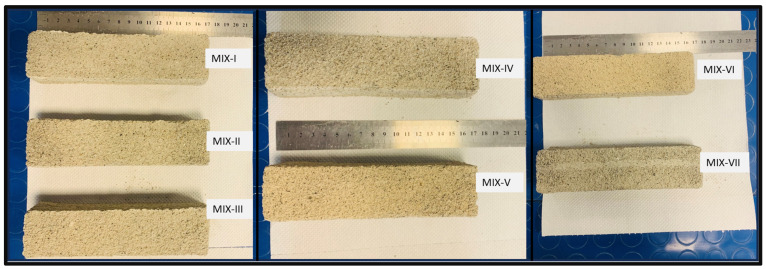
Macroscopic images of the samples produced using the different mixes.

The macroscopic pictures shown in Figure 2 provide important information about the appearance of the printed specimens and how they relate to the mix ratios that were used. One noteworthy finding is the considerable color variation among the specimens, which suggests discrete compositional variations brought about by dissimilarities in the mix ratio. This color variation highlights the sensitivity of additive manufacturing to minute changes in formulation. It may result from variations in material composition, density or chemical reactions occurring during the printing process. Additionally, nozzle blockage in the Mix-VII specimen is an important observation since it affects the final geometry’s finishing. Nozzle blockage can cause inconsistencies in layer deposition and reduced structural integrity by interfering with the extrusion process. This result emphasizes how crucial it is to optimize material compositions and printing conditions in order to reduce the possibility of printing errors and guarantee reliable print quality. Overall, the macroscopic examination of the printed specimens reveals opportunities for additive manufacturing process optimization and offers insightful qualitative information about the effects of mix ratio variations.

### 3.1. Flowability

The flowability of Mix-I to Mix-VII was measured and the data in Table 2 clearly show that the Hausner ratio and Carr’s index decrease together when one moves from Mix-I to Mix VII. As the mixtures’ composition changes, this descending pattern points to a gradual increase in flowability, offering important information on the packing and flow properties of the powder.

### 3.2. Density

**Figure 3:**
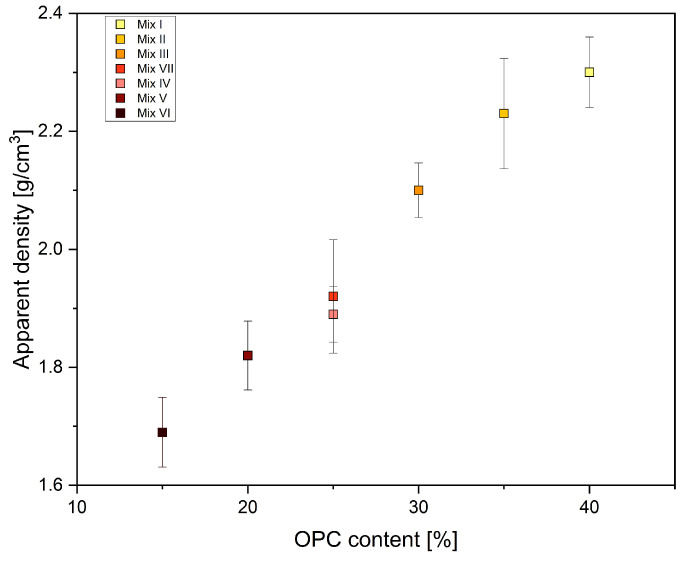
Impact of OPC content on the density of 3D-printed specimens.

Figure 3 shows the relationship between OPC content and apparent density. As the OPC content increases, it likely contributes to improved particle packing, leading to a more tight arrangement.

When OPC and quick-setting cement are combined, the increased density is still due to OPC’s higher specific gravity.

### 3.3. Mechanical Properties

**Figure 4:**
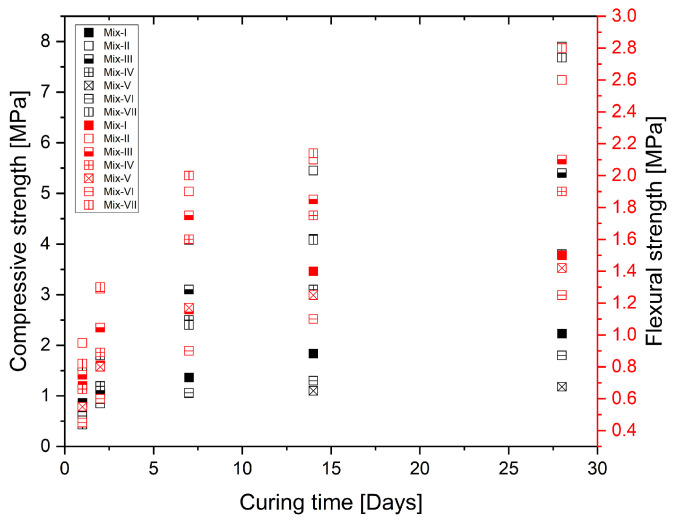
Mechanical properties of printed specimens.

Figure 4 shows the mechanical properties of the printed specimens. Throughout the course of the 28-day curing period, Mix-II shows a notable superiority in terms of compressive and flexural strengths. These compositions likely promote interfacial bonding within the concrete [61] and improved particle packing of the concrete matrix, which improves its mechanical properties. Another important factor for improved mechanical properties of the mixtures is the specific proportion of the ingredients that may result in superior hydration kinetics and improved microstructural development during the process. Furthermore, it is possible that the chemical interactions and phase transitions that occur within these mixtures during hydration play an important role in reinforcing the material structure and increasing mechanical performance over time.

**Figure 5:**
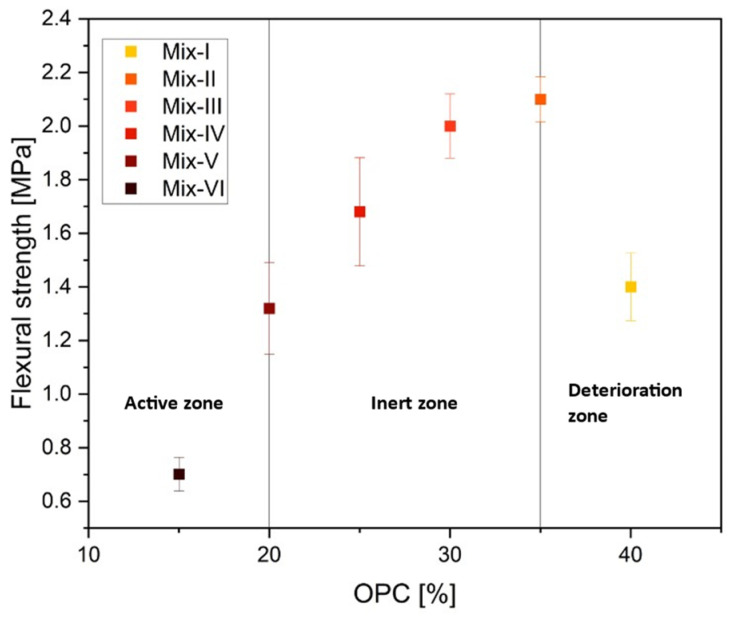
Behavior of 3D-printed specimens at varying OPC content across active, inert and deterioration zones after seven days of curing.

The mix proportions utilized in the trials to obtain the flexural strength findings are shown in Table 1. The results are classified into three zones [62] based on the different proportions of cement content, namely active, inert and deterioration zones. The active zone is the region where a larger cement content enhances bonding at grain contact points, leading to increased strength. In the inert zone, strength development gradually slows down at some level, and further addition of cement content decreases the strength instead of improving it, thus, the so-named deterioration zone (Figure 5). These results suggest that increasing the cement content beyond a certain limit may not contribute significantly to strength improvement and, for the binary mixture considered here, the limit corresponds to Mix-II.

**Figure 6:**
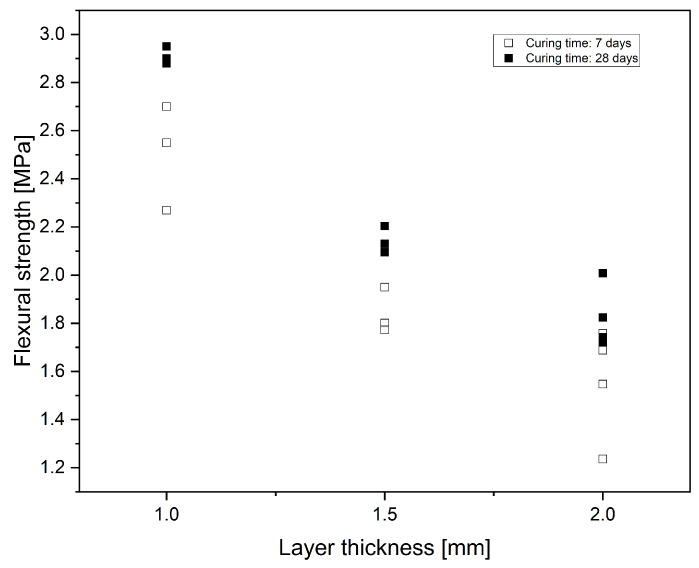
Flexural strength of 3D-printed specimens with layer thickness.

We observe an inverse proportionality between the mechanical strength and the printed layer thickness for Mix-VI, as shown in Figure 6. A reduced layer thickness appears to lead to more compact powder bed packing, stability and uniformity, with these contributing to higher strength.

The study identified limitations pertaining to powder size, layer thickness and flowability, all of which are essential for attaining the best possible outcome in additive manufacturing. Proper adhesion and compaction are ensured by keeping the powder size lower than the printed layer. Also, correct flowability is necessary for uniform distribution of powder on the bed.

The findings of this study have important significance for the construction sector, particularly in the optimization of concrete mixtures and additive manufacturing techniques. The study determined a particular mixture composition that demonstrated better mechanical qualities after 28 days of curing by thoroughly examining several combinations of binary mixtures. For professionals in the industry looking to improve the performance of concrete compositions, this finding provides insightful information. Furthermore, the layer thickness study demonstrated that lowering the thickness from 2 mm to 1 mm using concrete binder jetting resulted in increased performance.

### 3.4. Microscopic Analysis

**Figure 7:**
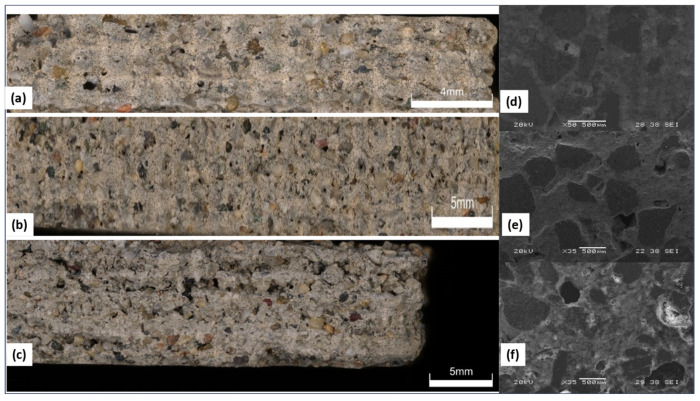
(**a**) Microscopic optical image at layer thickness = 1 mm, (**b**) layer thickness = 1.5 mm and (**c**) layer thickness = 2 mm. (**d**) SEM image at layer thickness = 1 mm, (**e**) layer thickness = 1.5 mm and (**f**) layer thickness = 2 mm.

To analyze further the impact of layer thickness, side-view images of printed specimens from the optical digital microscope and top surface SEM images of the polished specimens are shown in Figure 7. SEM images clearly show the voids that can also be observed at larger layer thickness. The images suggest that the reduced strength at higher layer thickness in Figure 6 is correlated to a larger number of voids.

Apart from the trends that have been noted in terms of void formation and mechanical strength, it is imperative to take into account the compaction behavior of the printed layers at different layer thickness. Since the wiper blade is applied consistently, the compaction of each layer becomes more uniform as the layer thickness decreases, resulting in more densely packed layers. Such improved compaction relies on the material being deposited over a reduced distance, which facilitates greater consolidation of the printed layers. On the other hand, specimens created at larger layer thicknesses can show less consistent compaction because of the possibility of uneven material distribution and compaction due to the increased distance between layers. Thus, decreasing the layer thickness leads to both a decrease in void formation and a greater degree of uniform compaction of the layers, which improves the mechanical strength. Future studies could investigate more sophisticated compaction methods and printing strategies to further optimize the compaction process and maximize the mechanical properties of printed concrete structures.

## 4. Conclusions

The study investigated the behavior of a modified binary cement mixture composed of ordinary Portland cement and quick-setting cement during printing with a customized binder jetting 3D printer.

This work leads to the following significant conclusions.

- The flow characteristics of mixtures with lower OPC content were good to fair, while those with higher OPC content showed passable to cohesive/poor flow. The Hausner ratio (HR) and Carr’s index (CI) show that flow dynamics were affected by the steady increase in OPC concentration. These results indicate the significance of OPC concentration in determining the general flow behavior of binary cement mixtures and provide important information for uses in 3D printing technology.
- The OPC content plays a critical role in improving the overall density of the 3D-printed specimens. As the proportion of OPC cement exceeds that of quick-setting cement, the higher specific gravity of OPC becomes the primary factor in improving the overall density and the mechanical properties of the material.
- Mix-VII exhibits relatively high flexural strength, peaking at 1.9 MPa after seven days and increasing to 2.7 MPa by the 28th day. Further highlighting Mix-VII’s strong mechanical performance is this positive trend in compressive strength over time.
- Variable binary cement content improves mechanical properties up to 35:5:60 wt% of OPC:QSC:sand; above that, more cement content causes the strength to decrease.
- The findings show that there is an apparent reduction in mechanical strength as layer thickness increases. This result implies that the mechanical strength and layer thickness are inversely related. This observed pattern emphasizes how crucial it is to take layer thickness into account as an essential.
- Microscopic analysis confirms a correlation between strength and voids at different layer thicknesses, with the increase in voids appearing as the source of the strength decrease. In conclusion, our research clearly shows how layer thickness affects void formation and mechanical strength in printed concrete samples.

## Figures and Tables

**Table 1:** Composition of the dry mixtures used in the BJ3DP process.

Mixture	Content (Sand:OPC:QSC) wt%
Mix-I	50:40:10
Mix-II	60:35:5
Mix-III	70:30:0
Mix-IV	70:25:5
Mix-V	70:20:10
Mix-VI	70:15:15
Mix-VII	75:25:0

**Table 2:** Flowability of dry powder mixtures.

Mixture	HR	CI	Flow Character
Mix-I	1.35	26	Cohesive/poor
Mix-II	1.31	24	Passable
Mix-III	1.28	22	Passable
Mix-IV	1.28	22	Passable
Mix-V	1.21	18	Fair
Mix-VI	1.17	15	Good
Mix-VII	1.23	19	Fair

## Data Availability

Data are contained within the article.
